# Supplementary material for: Inflammatory factors and risk of lung adenocarcinoma: a Mendelian randomization study mediated by blood metabolites
Source: Front Endocrinol (Lausanne). 2024 Aug 27;15:1446863. doi: 10.3389/fendo.2024.1446863 (PMC11384989; doi:10.3389/fendo.2024.1446863)
Supplement: Supplementary file 1 [file DataSheet1.zip › supplementary files/S2/GCST90200994/forest.pdf]

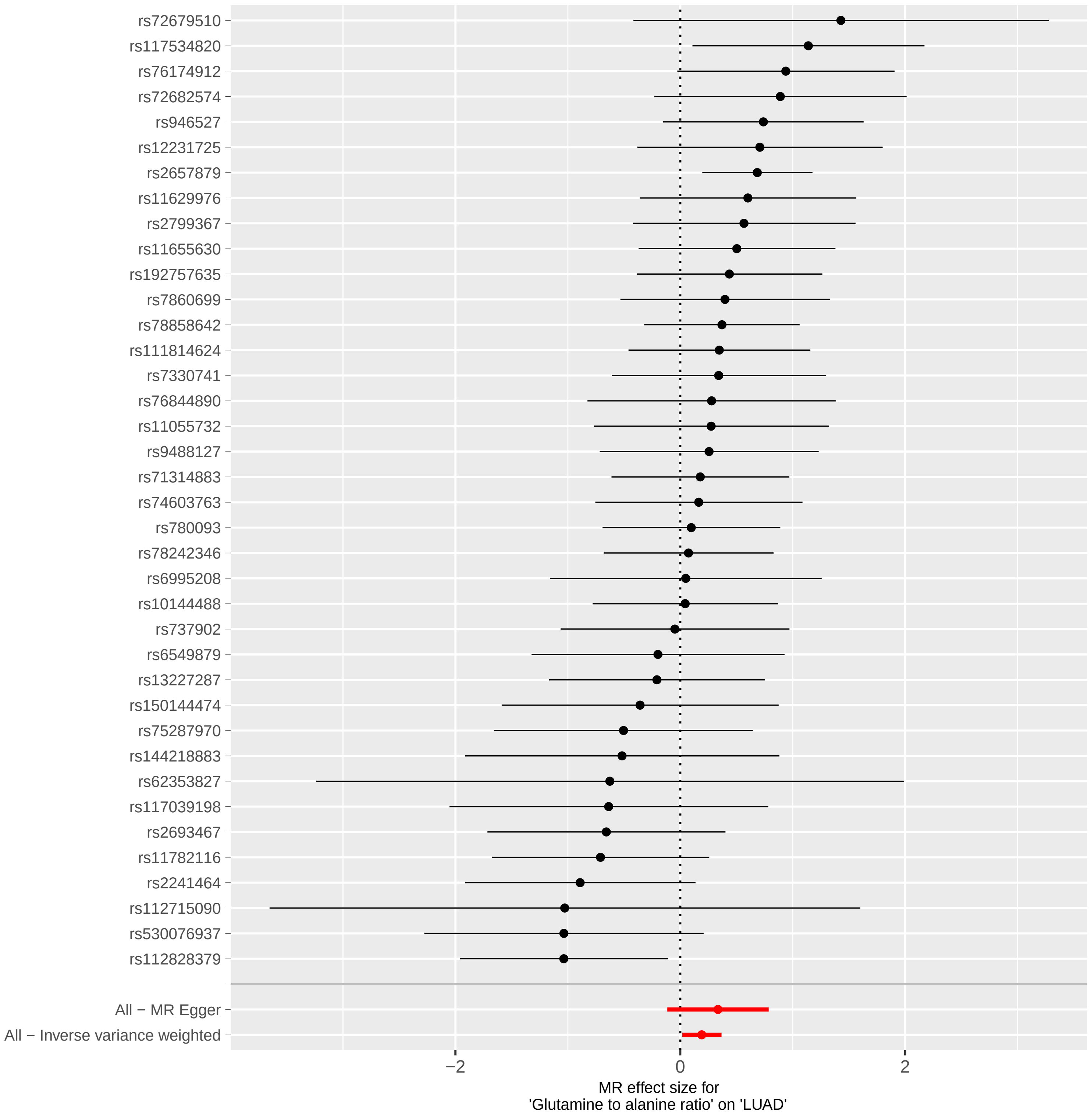
rs72679510
rs117534820
rs76174912
rs72682574
rs946527
rs12231725
rs2657879
rs11629976
rs2799367
rs11655630
rs192757635
rs7860699
rs78858642
rs111814624
rs7330741
rs76844890
rs11055732
rs9488127
rs71314883
rs74603763
rs780093
rs78242346
rs6995208
rs10144488
rs737902
rs6549879
rs13227287
rs150144474
rs75287970
rs144218883
rs62353827
rs117039198
rs2693467
rs11782116
rs2241464
rs112715090
rs530076937
rs112828379
All − MR Egger
All − Inverse variance weighted
−2
0
2
MR effect size for
'Glutamine to alanine ratio' on 'LUAD'
